# The Citius End: World Records Progression Announces the Completion of a Brief Ultra-Physiological Quest

**DOI:** 10.1371/journal.pone.0001552

**Published:** 2008-02-06

**Authors:** Geoffroy Berthelot, Valérie Thibault, Muriel Tafflet, Sylvie Escolano, Nour El Helou, Xavier Jouven, Olivier Hermine, Jean-François Toussaint

**Affiliations:** 1 Institute for Biomedical Research and Sports Epidemiology (IRMES), Paris, France; 2 INSERM, IFR69, U780, Villejuif, France; 3 Université Paris-Descartes, Paris, France; 4 Centre National de la Recherche Scientifique (CNRS) UMR 8147, Hôpital Necker, Paris, France; 5 Centre d'Investigation en Médecine du Sport (CIMS), Hôtel-Dieu, Assistance Publique-Hôpitaux de Paris (AP-HP), Paris, France; Universidad Europea de Madrid, Spain

## Abstract

World records (WR) in sports illustrate the ultimate expression of human integrated muscle biology, through speed or strength performances. Analysis and prediction of man's physiological boundaries in sports and impact of external (historical or environmental) conditions on WR occurrence are subject to scientific controversy. Based on the analysis of 3263 WR established for all quantifiable official contests since the first Olympic Games, we show here that WR progression rate follows a piecewise exponential decaying pattern with very high accuracy (mean adjusted *r^2^* values = 0.91±0.08 (s.d.)). Starting at 75% of their estimated asymptotic values in 1896, WR have now reached 99%, and, present conditions prevailing, half of all WR will not be improved by more than 0,05% in 2027. Our model, which may be used to compare future athletic performances or assess the impact of international antidoping policies, forecasts that human species' physiological frontiers will be reached in one generation. This will have an impact on the future conditions of athlete training and on the organization of competitions. It may also alter the Olympic motto and spirit.

## Introduction

**Figure 1:**
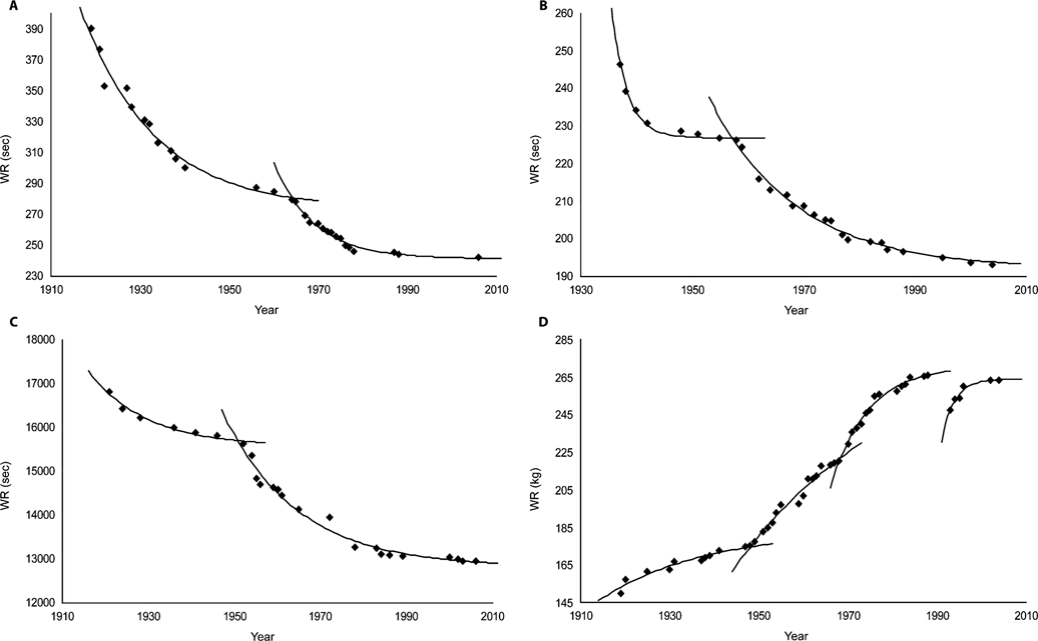
Model fitting on 4 events. A. Women 400 m freestyle (swimming) with biexponential decaying curve, adjusted *r_i_^2^* = 0.976 and *r_ii_^2^* = 0.966; B. Men 4×100 m freestyle relay (swimming), *r_i_^2^* = 0.985, *r_ii_^2^* = 0.988; C. Men 50 km walk (track), *r_i_^2^* = 0.972, *r_ii_^2^* = 0.977; D. Clean & Jerk Super Heavyweight (weight lifting), *r_i_^2^* = 0.939, *r_ii_^2^* = 0.937, *r_iii_^2^* = 0.975 and *r_iv_^2^* = 0.946. Weight categories were altered in 1948, 1968 and 1992 and control reinforced in 1988–1992 in weight lifting.

Olympic Games were reintroduced in 1896 by Pierre de Coubertin. One hundred and eleven years later, world record collection shows the progression of human performance as elite athletes periodically pushed back the frontiers of “ultra-physiology”. This unplanned experiment could have been written as the phenotypic maximization of present human genotype under the pressure of regulated competition [1]. This large scale investigation can now be appraised, but the best methodology to do it is a disputed scientific issue [2]–[5], with some literary perspectives [6], [7]. Linear regression models [2], [8] have been criticized [3] for their inaccuracy and non physiological relevance. A flattened S-shaped model has been elaborated by Nevill and Whyte [4], [5] on 8 running and 6 swimming events, but closer observation would suggest more detailed variations of the WR curves, adding historical or technical influences to biological parameters (Fig. 1). Here we identify a common progression pattern for world records from all quantifiable Olympic events and propose a model that predicts the end of the quest.

## Materials and Methods

We conducted a qualitative and quantitative analysis of 3263 WR in all 147 measurable Olympic events from five disciplines [9]–[13] in order to identify WR progression patterns. Data were gathered from 1896 to 2007 (modern Olympic era).

### Descriptive analysis: *λ, κ* factors

Two indicators were introduced in order to describe WR. Because the WR number established each year also depends on the number of events, we defined factor *λ* as the annual ratio at year *t* of the new WR number over the total number of official Olympic events:
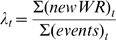
(1)WR evolution is also analyzed through the progression step *κ*, which measures the relative improvement of the *n* ^th^ best performance as compared to the *n-1* ^th^ value:
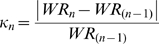
(2)with a mean *κ* *_t_* annually calculated for all official events at year *t*.

### Function description

WR series for each event were fitted by the function
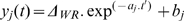
(3)


Where Δ*_WR_* = *WR_i,j_*−*WR_f,j_* is an event indicator for the studied *j* period; it is positive for the chronometric events (with decreasing WR values) and negative for the non-chronometric ones (increasing WR values); *WR_i,j_* and *WR_f,j_* are the initial and final WR values, respectively ; *a_j_* is the positive curvature factor given by non linear regression; *b_j_* is the asymptotic limit. Normalization of *t* in the [0, 1] interval ensures the objective function (3) to be well-defined for all values of *t*. As a consequence, we used:
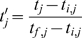
(4)where *t'* is the WR year after linear transformation of *t* ; *t_i,j_* and *t_f,j_* are the years of initial and final WR in the current *j* period, respectively. Equation (3) assumes that WR will achieve an asymptotic value within a given span starting at WR_i,j_.

### Splitting WR series into periods

A procedure based on the best adjusted *r^2^* is used to split WR series into periods. The algorithm is initiated by the first three WR values. The series is iteratively fitted by adding the next WR point using equation (3). For each fit, the adjusted *r^2^* is obtained; local maxima provide the changes of incline corresponding to the beginning of a new period. The minimum period duration is 6 years, the minimal WR number is three per period.

For each event, this piecewise exponential decaying model provides successive periods. A period refers to a time slot defined by a group of consecutive WR, following a rupture of incline. During the period *j*, parameters *a_j_* and *b_j_* are estimated using the Levenberg-Marquardt algorithm [14]–[16] (LMA) in a non linear least-squares regression to fit the model to WR. High values of the curvature coefficient *a* are seen in highly curved periods showing weak margin of final progression. Coefficient *b* is the asymptotic value; the comparison of the initial (WR_i_) and final (WR_f_) records to *b* are described through the *b′* and *b″* ratios respectively. The progression step over the Olympic era is equal to *b″*–*b′* and expressed as a percentage of the asymptotic value.

### Coefficients description

The initial progression range is given by *b′*. In order to compare the predicted final progression, *b″* are calculated for terminal periods of events.

For chronometric events (WR_i_>WR_f_): 
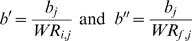
; for non chronometric events (WR_i_<WR_f_): 
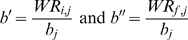
.

This presentation also allows for a comparison of each record as a percentage of the estimated asymptotic value.

### Prediction

Data set used for prediction was reduced to 125 events. From the 22 discarded events, two resulted from javelin weight change and 20 referred to weight lifting: 9 Clean and Press events were removed from official list in 1972 (Fig. S2) and 11 suffered major rule's alterations (weight categories). For prediction purpose, the inverse function of equation (3), is given by:
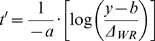
(5)


Coefficients of equation (5) are calculated for the last period of each event through LMA. The result is used to estimate the year *t* when the 99.95% (1/2000) limit is reached. This limit is set by 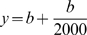 for chronometric events, and 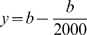 for non chronometric records. The 1/2000 value was chosen in reference to the chronometric limits used on the quickest track race: it represents half of a 1/100^th^ of a second on the 100m (and about 4s on a marathon or 100g in weight lifting disciplines).

### Estimating prediction variability

Credibility interval is computed using a simulation method of Monte Carlo [17]. Previously estimated coefficients from equation (5) are used to draw 10 000 new coefficients in a bidimensional normal distribution. Median was chosen as a robust measure of the center of the distribution in a non parametric approach.

We used the 2.5th percentile, median and 97.5th percentile to produce the prediction errors for the estimated year at 99.95% and the estimated WR asymptotic values. The credibility interval [18] is given by the mean of the 2.5th and 97.5th percentiles for all 125 predictable events (Table S1).

## Results

Chronometric events represent 58% of the data set (swimming, track, cycling, speed skating) with a decreasing tendency of WR values; 42% are non chronometric events (field, weight lifting) with increasing WR values.

**Figure 2:**
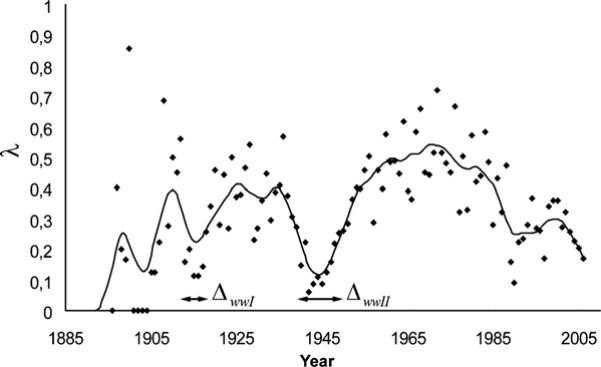
Evolution of factor *λ* : new WR number over official Olympic event number. Exact numbers (black dots) are filtered with a 60 Hz second order low pass butterworth filter (black curve). World wars show major impact on *λ* : Δ*_wwI_* = 6.4 years ; Δ*_wwII_* = 13.4 years.

Factor *λ* evolution during the Olympic era (Fig. 2) reveals three major phases of decline starting in 1913, 1938 and 1971 respectively. World Wars impact on *λ* results in two major lag times, estimated by their width at mid-height: Δ*_wwI_* = 6.4 years for World War I and Δ*_wwII_* = 13.4 years for World War II. The calculated mean delay between each new WR is 2.62±3.05 (s.d.) years.

**Figure 3:**
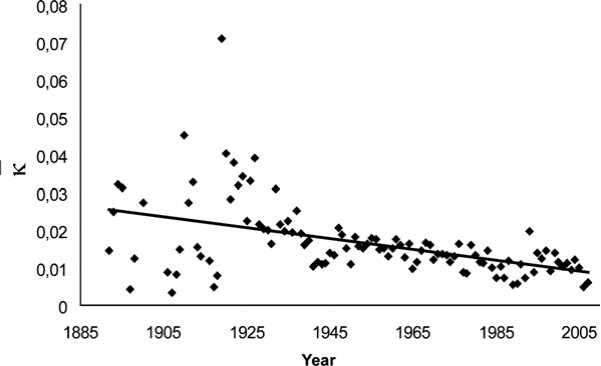
Annual evolution of WR relative improvement: *κ* decreases from 0.024 in the first 30 years to 0.010 in the last 10 years (Linear model: *y* = −1.46·10^−4^ *x*+0.301, *F*(1,102) = 27.14, *P*<0.001). This decrease is representative of the growing difficulty to improve previously established WR values.

*κ* *_t_* significantly diminishes over the whole studied era (linear model: *F*(1,102) = 27.14, *P*<0.001) (Fig. 3) supporting the hypothesis of a constant reduction of WR progression possibilities.

Using the best adjusted *r^2^* iterative algorithm, 363 periods were obtained with mean *a* = 3.00±2.87, *b′ = *0.75±0.15 (Fig. S4), *b″* = 0.99±0.008, an average progression step between initial and final records of 24%, and mean adjusted *r^2^* = 0.91±0.08 (Fig. S3). The evolutionary profile of WR series over the Olympic era shows 2 to 3 periods for most of the events (2.47 periods±1.18, range 1–6), with a mean of 8.98 WR per period and a mean duration of 25.8 years±14.8 per period.

**Figure 4:**
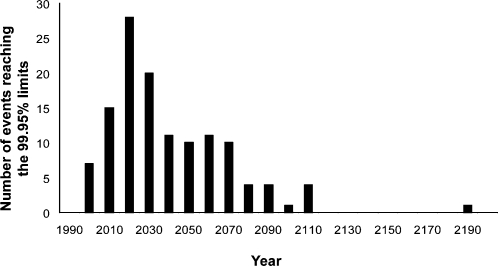
Distribution of estimated limits at 99.95% of the asymptotic value. Results are sampled by decades. Half of the asymptotic records will be established in 2027, and 90% in 2068.

We predicted the asymptotic value of each record, using the inverse function of equation (3) on the last period of 125 exploitable events (Table S1). A Monte-Carlo procedure was used to define the credibility interval of the prediction. The mean credibility interval of the asymptotic WR values is [−2.28% ; +2.28%]. We also predicted the year when a record will be established at 99.95% of its asymptotic value using the same method on the last period of the 125 exploitable events. The distribution of the 125 dates is expressed by decades (Fig. 4): 12.8% of these asymptotic WR have been reached in 2007. By 2027, half of the records will reach 99.95% of their asymptotic value, within a [2002–2120] credibility interval (Table S1 for each event prediction).

## Discussion

The proposed piecewise exponential decaying model, describing momentary expansion in a finite context, suggests a major global fading of WR progression. During an initial phase of rapid improvement, interrupted by two major events (Fig. S1), WR progression rate may have been described by a linear model. With a 40 years hindsight on the WR rate decline (Fig. 2), debate on the limits now clearly emerges. As expected from biology, accurately fitted curves (high *r^2^* values) now refute the linear model. In all measurable Olympic contests from five different disciplines, involving either aerobic (10000 m skating) or anaerobic (weight lifting) metabolic pathways, leg muscles mainly (cycling) or all muscles (decathlon), lasting seconds (shots) or hours (50 km walk), either in men or women, small (Fly weight) or tall athletes (100 m free style), individual or collective events (relays), all progression curves follow the same pattern, supporting the universality of the model.

Recently introduced events, such as women weight lifting starting in 1998 (Fig. S2), may require closer follow-up. Also final periods appearing in the last decade, with smaller data samples, may have a wider progression margin than estimated. Record measurement accuracy may be enhanced by using more precise technologies (times recorded in milliseconds, jumps in millimeters). Such decisions, however, are expected to have no effect on the WR progression rate *λ*, as it only alters the curve sampling and not the exponential shape nor the asymptotic value.

Historical circumstances and WR evolution are closely linked: the impact of world wars results in two delays (Fig. 2) with Δ*_wwII_* being twice as large as Δ*_wwI_*. Starting in 1971, a much larger *λ* reduction is observed (from 0.72 to 0.17), in the absence of major conflict [19] and despite the Cold War, which boosted sport competition among east European and western nations. In addition economic development between 1950 and 1980, with major technological, nutritional and medical advances, offered a constant elevation of life resources in the few countries (USA, Russia, Australia, Canada, Japan and European countries) that provide 95% of WR. This last WR rate decrease also happened despite a considerable expansion of participating countries (and athletes) : from 14 nations in 1896 in Athens (240 competitors), 69 nations in Helsinki's 1952 games (4950 athletes) and 202 nations for the last summer games in Athens again (11100 athletes). Rule modifications and antidoping control reinforcements may have generated specific WR evolutions, as in Clean & Jerk super heavyweight (Fig 1.D), where weight categories changed in 1948, 1968 and 1992. Finally, the *λ* decrease still appeared despite improvement of selection and training processes (time allotted to practice, new jumping or race-starting techniques, recruitment of taller athletes [20]). All of these may have triggered new periods, but did not alter the global pattern, which is common to sport events as different as marathon, 4×100 m medley relay or pole vault.

Individual or team doping strategies have been used throughout the Olympic era, and state controlled protocols were developed since 1970 [21], [22] : both may have contributed to slow down the *λ* slope. Such practices however did not prevent the decline observed after the Mexico Games. Situations where doping would be legalized or not properly prevented [23]–[26] may again partially alter the record course in the future. The fact that suspected or belatedly convicted athletes hold some of the final records may exaggerate our predictive model, such that the year when half of WR will reach the 99.95% limit may even be closer. In fact recent data show no progression of the 10 best performers in the last 20 years for the 100 m track women or men high jump [27] suggesting these WR may not be challenged anymore, especially when anti-doping agencies increase their actions and penalties. This is also observed when comparing the sprint events in running, swimming and speed skating over the second half of the XX^th^ century [28].

Major international rule changes, new technologies or gene profiling have also been tested in sport [29]–[30]. However 50% of asymptotic WR values will be obtained in one generation: sport organizations may then try to create new events, drop the WR quest, favor sports less directly associated with pure performance or promote health benefits of physical activity [31]. The “citius, altius, fortius” motto may be reworded within this century. Toward a “sanius ?” remains an open question.

In summary, an epidemiological analysis of sport performances demonstrates that WR progression follows a piecewise exponential decaying pattern, altered by historical events. Results point out that in 2007, WR have reached 99% of their asymptotic value. Present conditions prevailing for the next 20 years, half of all WR won't be improved by more than 0.05%. As compared to the positivism triumphing at the time Coubertin inspired Olympic renewal, the present analysis emphasizes the ineluctable rarefaction of the quantifiable proofs of human physiological progression.

## Supporting Information

Figure S1 Evolution of cumulative annual number of records. The growth of WR is altered by the two world wars, and is slowing down since 1988.
(0.15 MB TIF)

Figure S2 Evolution of cumulative annual number of events with official WR. In 1972, 9 weight-lifting events were discarded from Olympic event list; 14 women weight lifting events were introduced in 1998.
(0.15 MB TIF)

Figure S3 Adjusted r^2^ values for the 363 periods showing no variation over the modern Olympic era (Linear model: F(1,361) = 1.268, P = 0.261) and a mean value of 0.91±0.08.
(0.20 MB TIF)

Figure S4 Evolution of b': this parameter increases (Linear model: F(1,145) = 106.7, P<0.001) during the Olympic era, such that recently introduced events will reach their asymptote faster than early XXth century contests.
(0.17 MB TIF)

Table S1 Table of predicted WR asymptotic value, year of the 99.95% limit, credibility intervals and period number per event. Women and men are respectively symbolized by (W) and (M). C&J are Clean and Jerk weight lifting events. Two versions of the Track women 100 m are presented, version 1 includes the last WR, version 2 leaves it out.
(0.29 MB DOC)
