# Supplementary figures and images for: The Citius End: World Records Progression Announces the Completion of a Brief Ultra-Physiological Quest
Source: PLoS One. 2008 Feb 6;3(2):e1552. doi: 10.1371/journal.pone.0001552 (PMC2212132; doi:10.1371/journal.pone.0001552)

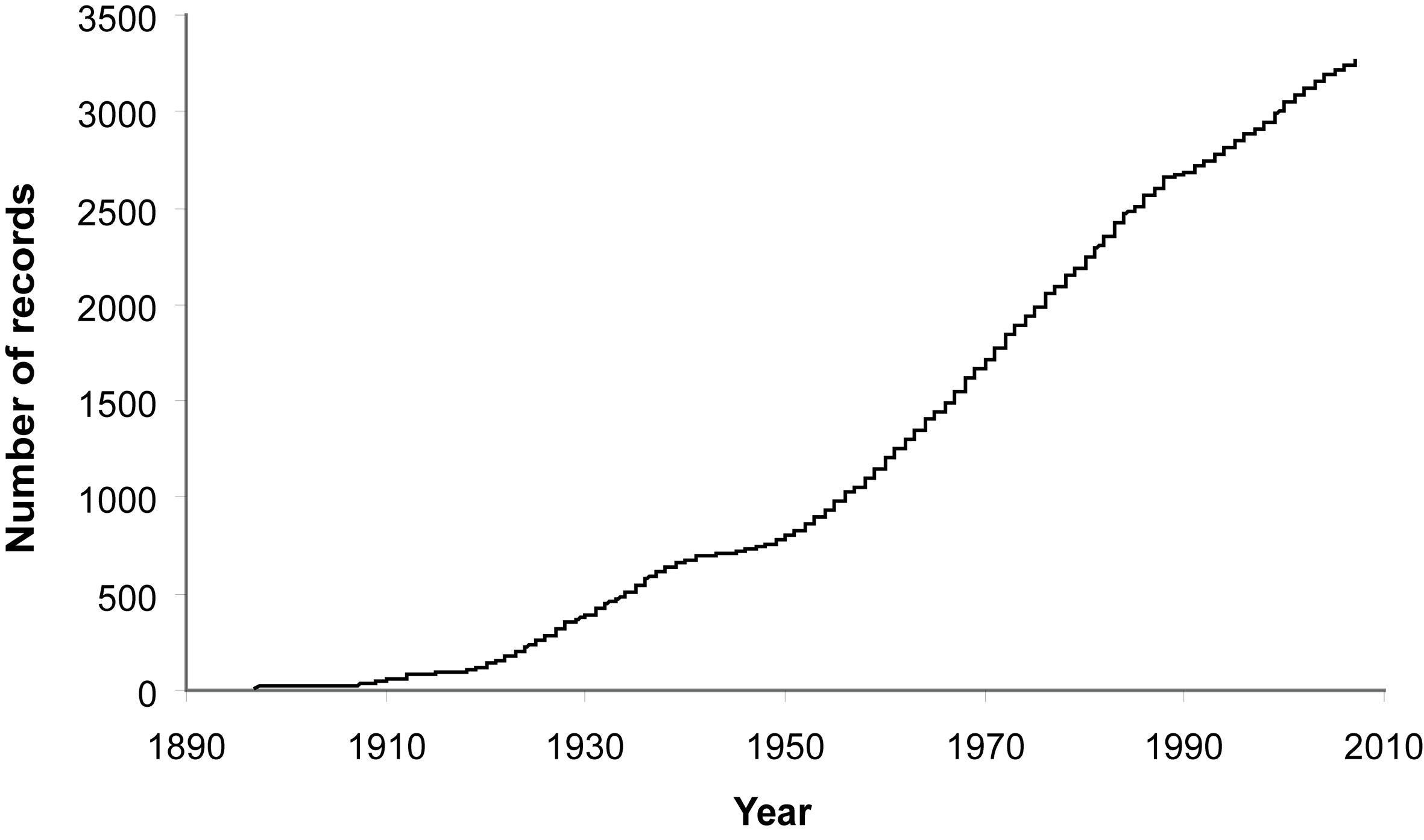

Supplement: Figure S1 — Evolution of cumulative annual number of records. The growth of WR is altered by the two world wars, and is slowing down since 1988. (0.15 MB TIF) [file pone.0001552.s001.tif]

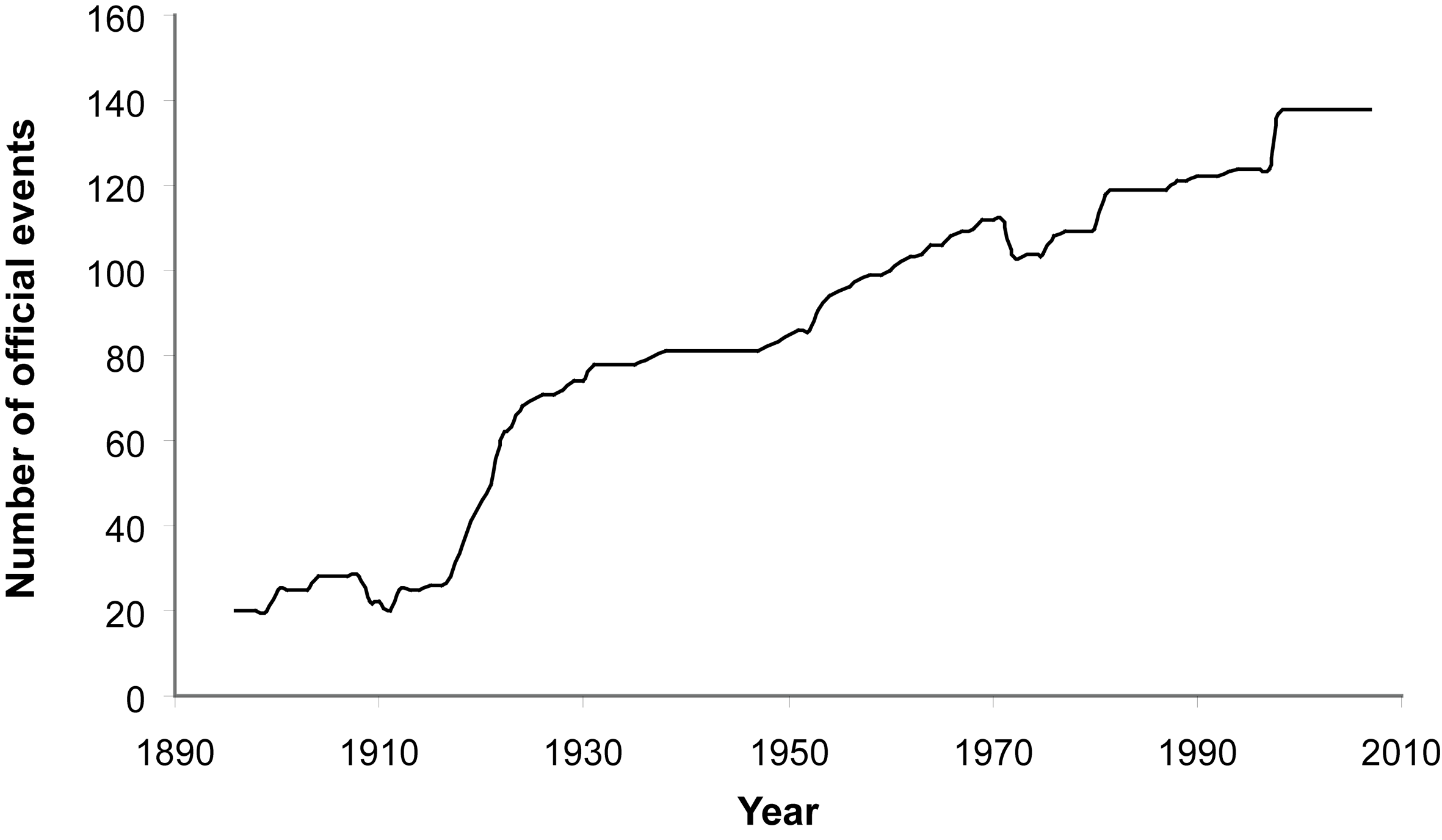

Supplement: Figure S2 — Evolution of cumulative annual number of events with official WR. In 1972, 9 weight-lifting events were discarded from Olympic event list; 14 women weight lifting events were introduced in 1998. (0.15 MB TIF) [file pone.0001552.s002.tif]

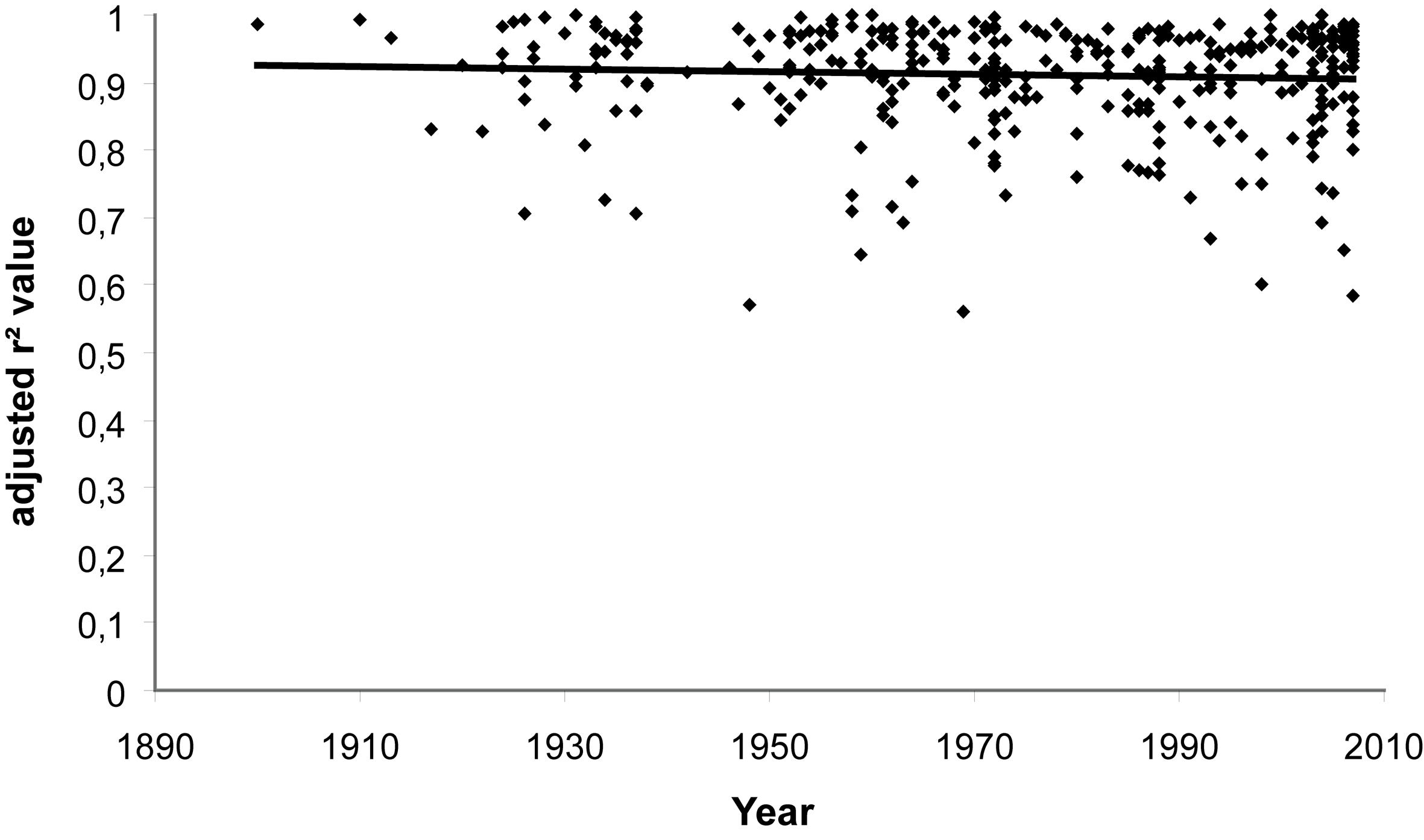

Supplement: Figure S3 — Adjusted r2 values for the 363 periods showing no variation over the modern Olympic era (Linear model: F(1,361) = 1.268, P = 0.261) and a mean value of 0.91±0.08. (0.20 MB TIF) [file pone.0001552.s003.tif]

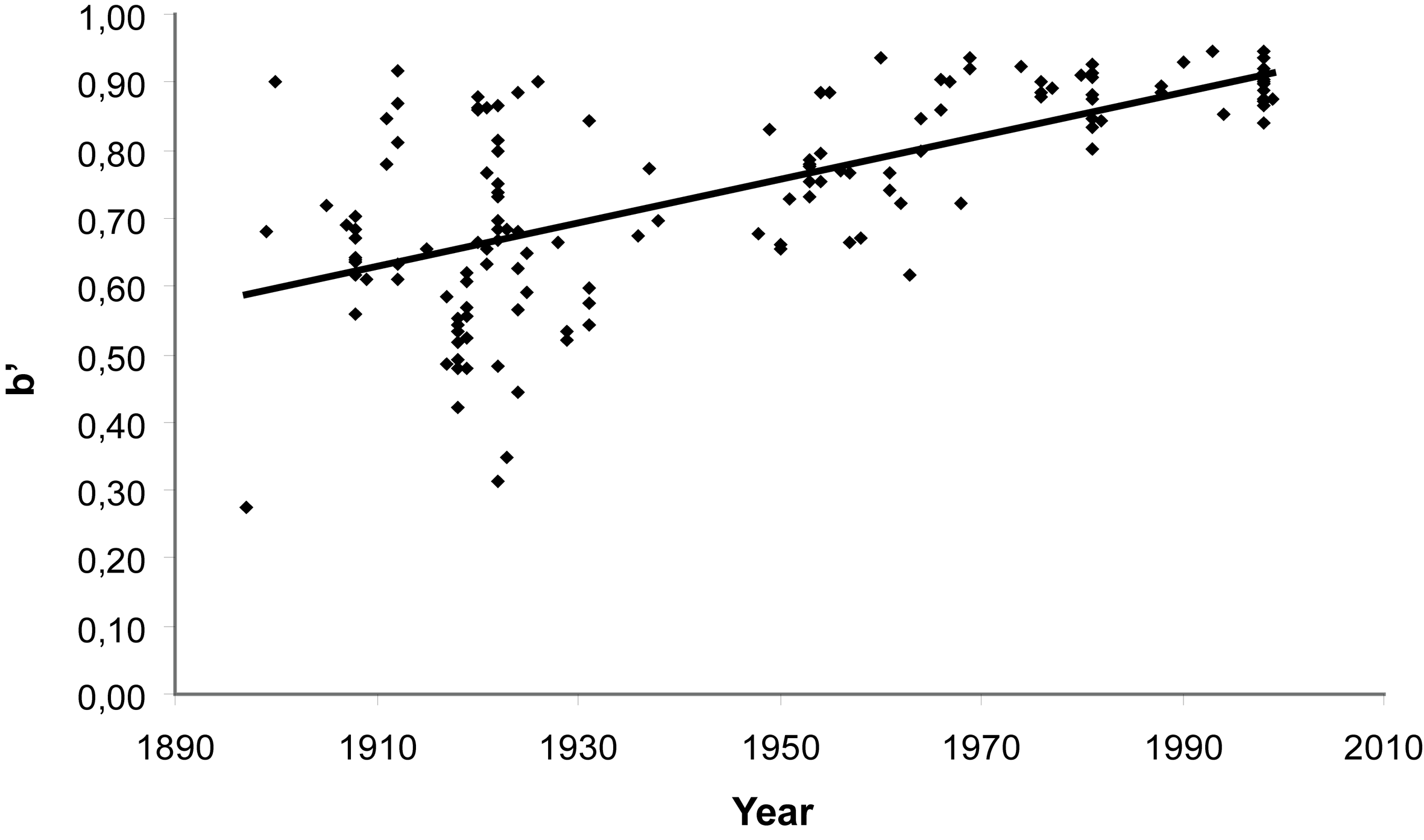

Supplement: Figure S4 — Evolution of b': this parameter increases (Linear model: F(1,145) = 106.7, P<0.001) during the Olympic era, such that recently introduced events will reach their asymptote faster than early XXth century contests. (0.17 MB TIF) [file pone.0001552.s004.tif]
