# Supplementary material for: The Citius End: World Records Progression Announces the Completion of a Brief Ultra-Physiological Quest
Source: PLoS One. 2008 Feb 6;3(2):e1552. doi: 10.1371/journal.pone.0001552 (PMC2212132; doi:10.1371/journal.pone.0001552)
Supplement: Table S1 — Table of predicted WR asymptotic value, year of the 99.95% limit, credibility intervals and period number per event. Women and men are respectively symbolized by (W) and (M). C&J are Clean and Jerk weight lifting events. Two versions of the Track women 100 m are presented, version 1 includes the last WR, version 2 leaves it out. (0.29 MB DOC) [file pone.0001552.s005.doc]

|  | *Asympt* | *Predicted Interval* | | *Year of* | *Credibility* | | *Period* |
| --- | --- | --- | --- | --- | --- | --- | --- |
| *Events* | *value* | *variation from asympt* | | *99,95%* | *Interval* | | *number* |
| Cycling - 500m Time Trial (W) | 33,289s | 32,483 | 34,084 | 2034,6 | 2002,2 | 2176,6 | 1 |
| Cycling - Individual Pursuit (W) | 203,272s | 194,021 | 212,627 | 2015,8 | 1995,8 | 2580,5 | 2 |
| Cycling - Sprint (W) | 10,689s | 10,347 | 11,033 | 2031,9 | 1994,3 | 2163,1 | 1 |
| Cycling - 1km Time Trial (M) | 57,944s | 55,721 | 60,196 | 2064,5 | 2000,6 | 2318,5 | 3 |
| Cycling - Individual Pursuit (M) | 247,298s | 228,227 | 266,962 | 2026,0 | 1986,3 | 2580,2 | 2 |
| Cycling - Sprint (M) | 9,728s | 9,676 | 9,778 | 2025,4 | 2012,5 | 2044,0 | 2 |
| Cycling - Team Pursuit (M) | 234,582s | 230,940 | 238,277 | 2030,5 | 2005,7 | 2098,3 | 2 |
| Speed Skating - 500m (W) | 37,019s | 36,998 | 37,040 | 2007,5 | 2005,1 | 2010,4 | 3 |
| Speed Skating - 1000m (W) | 72,817s | 72,285 | 73,353 | 2016,6 | 2005,1 | 2045,2 | 4 |
| Speed Skating - 1500m (W) | 108,164s | 104,731 | 111,538 | 2099,0 | 2051,3 | 2177,1 | 3 |
| Speed Skating - 3000m (W) | 224,599s | 217,443 | 231,723 | 2100,6 | 2057,1 | 2167,2 | 2 |
| Speed Skating - 5000m (W) | 397,708s | 389,901 | 405,567 | 2056,5 | 2028,2 | 2100,2 | 2 |
| Speed Skating - Short Track 500m (W) | 42,119s | 41,149 | 43,068 | 2060,2 | 2037,9 | 2092,4 | 1 |
| Speed Skating - Short Track 1000m (W) | 88,493s | 86,568 | 90,436 | 2046,8 | 2023,1 | 2084,0 | 1 |
| Speed Skating - Short Track 1500m (W) | 136,956s | 133,085 | 140,879 | 2023,9 | 2004,9 | 2078,6 | 2 |
| Speed Skating - 3000m relay (W) | 249,876s | 246,991 | 252,740 | 2014,9 | 2004,6 | 2036,2 | 2 |
| Speed Skating - 500m (M) | 33,771s | 32,607 | 34,936 | 2074,1 | 2007,1 | 2268,7 | 5 |
| Speed Skating - 1000m (M) | 66,838s | 66,461 | 67,216 | 2009,5 | 2003,9 | 2019,4 | 3 |
| Speed Skating - 1500m (M) | 101,888s | 100,894 | 102,874 | 2017,5 | 2006,0 | 2046,1 | 6 |
| Speed Skating - 5000m (M) | 350,716s | 331,245 | 370,072 | 2181,5 | 2085,5 | 2350,9 | 3 |
| Speed Skating - 10000m (M) | 758,138s | 738,967 | 777,388 | 2023,0 | 1999,8 | 2369,4 | 4 |
| Speed Skating - Short Track 500m (M) | 40,944s | 40,443 | 41,452 | 2014,4 | 2002,1 | 2050,8 | 2 |
| Speed Skating - Short Track 1000m (M) | 83,426s | 80,996 | 85,895 | 2042,6 | 2007,7 | 2144,0 | 2 |
| Speed Skating - Short Track 1500m (M) | 128,816s | 124,884 | 132,790 | 2028,3 | 2004,4 | 2098,6 | 2 |
| Speed Skating - 5000m relay (M) | 395,775s | 386,571 | 405,085 | 2034,2 | 2009,0 | 2090,7 | 1 |
| Swimming - 50m free style (W) | 23,987s | 23,517 | 24,456 | 2019,4 | 1993,1 | 2139,0 | 2 |
| Swimming - 100m free style (W) | 53,108s | 52,539 | 53,673 | 2034,3 | 1998,5 | 2175,9 | 5 |
| Swimming - 200m free style (W) | 115,176s | 114,627 | 115,737 | 2027,3 | 2011,0 | 2050,5 | 2 |
| Swimming - 400m free style (W) | 241,906s | 241,749 | 242,063 | 2010,9 | 2006,6 | 2015,6 | 2 |
| Swimming - 800m free style (W) | 485,965s | 477,137 | 494,864 | 2036,2 | 2014,4 | 2065,5 | 2 |
| Swimming - 1500m free style (W) | 939,524s | 937,824 | 941,246 | 2027,2 | 2021,8 | 2032,8 | 3 |
| Swimming - 100m backstroke (W) | 59,331s | 59,162 | 59,499 | 2019,2 | 2007,9 | 2034,8 | 4 |
| Swimming - 200m backstroke (W) | 124,669s | 122,821 | 126,562 | 2032,6 | 2012,2 | 2063,1 | 3 |
| Swimming - 100m breaststroke (W) | 63,832s | 62,612 | 65,038 | 2104,3 | 2056,9 | 2176,4 | 3 |
| Swimming - 200m breaststroke (W) | 138,147s | 136,776 | 139,525 | 2088,4 | 2067,5 | 2113,7 | 3 |
| Swimming - 100m butterfly (W) | 55,833s | 54,126 | 57,530 | 2053,3 | 1997,8 | 2240,1 | 3 |
| Swimming - 200m butterfly (W) | 125,051s | 124,389 | 125,721 | 2046,0 | 1998,8 | 2212,3 | 2 |
| Swimming - 200m medley (W) | 129,067s | 127,814 | 130,295 | 2017,5 | 1998,3 | 2051,6 | 3 |
| Swimming - 400m medley (W) | 272,397s | 271,859 | 272,924 | 2020,0 | 2012,3 | 2028,9 | 2 |
| Swimming - 4*100m medley relay (W) | 233,449s | 230,630 | 236,306 | 2051,1 | 2025,8 | 2088,8 | 2 |
| Swimming - 4*100m freestyle relay (W) | 215,063s | 214,324 | 215,805 | 2024,6 | 2015,0 | 2036,0 | 3 |
| Swimming - 50m free style (M) | 21,5s | 21,338 | 21,659 | 2022,4 | 2009,7 | 2039,2 | 1 |
| Swimming - 100m free style (M) | 47,353s | 46,806 | 47,899 | 2054,8 | 2025,9 | 2094,2 | 2 |
| Swimming - 200m free style (M) | 103,445s | 101,607 | 105,272 | 2034,4 | 1991,2 | 2308,5 | 4 |
| Swimming - 400m free style (M) | 216,389s | 214,462 | 218,304 | 2069,4 | 2047,8 | 2096,8 | 4 |
| Swimming - 800m free style (M) | 454,336s | 451,785 | 456,907 | 2045,7 | 2033,3 | 2060,5 | 5 |
| Swimming - 1500m free style (M) | 870,014s | 866,048 | 873,946 | 2026,9 | 2017,1 | 2038,7 | 3 |
| Swimming - 100m backstroke (M) | 52,228s | 51,730 | 52,727 | 2080,4 | 2054,0 | 2115,6 | 3 |
| Swimming - 200m backstroke (M) | 112,446s | 111,164 | 113,748 | 2080,1 | 2047,0 | 2129,0 | 3 |
| Swimming - 100m breaststroke (M) | 57,991s | 57,223 | 58,758 | 2101,6 | 2064,7 | 2153,8 | 3 |
| Swimming - 200m breaststroke (M) | 126,91s | 125,293 | 128,520 | 2062,9 | 2033,9 | 2104,7 | 4 |
| Swimming - 100m butterfly (M) | 50,16s | 49,233 | 51,069 | 2015,2 | 2001,0 | 2133,8 | 3 |
| Swimming - 200m butterfly (M) | 112,462s | 111,465 | 113,483 | 2054,1 | 2041,6 | 2068,2 | 1 |
| Swimming - 200m medley (M) | 113,67s | 112,398 | 114,954 | 2067,9 | 2035,8 | 2118,3 | 2 |
| Swimming - 400m medley (M) | 242,685s | 240,830 | 244,544 | 2072,9 | 2060,7 | 2086,4 | 1 |
| Swimming - 4*100m medley relay (M) | 208,407s | 206,508 | 210,314 | 2065,5 | 2042,3 | 2097,4 | 2 |
| Swimming - 4*100m freestyle relay (M) | 190,918s | 190,062 | 191,777 | 2058,3 | 2048,2 | 2069,4 | 2 |
| Swimming - 4*200m freestyle relay (M) | 418,482s | 413,795 | 423,125 | 2072,6 | 2036,3 | 2130,1 | 3 |
| Track & Field - 100m (W) - version 1 | 10,426s | 10,007 | 10,841 | 2013,0 | 1974,1 | 2754,4 | 3 |
| Track & Field - 100m (W) - version 2 | 10,727s | 10,617 | 10,837 | 1996,3 | 1978,2 | 2066,1 | 3 |
| Track & Field - 200m (W) | 21,111s | 20,510 | 21,711 | 2054,7 | 1986,6 | 2277,3 | 3 |
| Track & Field - 400m (W) | 47,119s | 46,252 | 48,003 | 2016,3 | 1989,6 | 2076,1 | 2 |
| Track & Field - 800m (W) | 110,285s | 107,269 | 113,313 | 2061,8 | 2016,6 | 2139,9 | 2 |
| Track & Field - 1500m (W) | 230,181s | 229,907 | 230,457 | 1998,0 | 1992,8 | 2004,4 | 1 |
| Track & Field - 5000m (W) | 847,533s | 835,598 | 859,742 | 2060,0 | 2021,7 | 2134,9 | 1 |
| Track & Field - 10000m (W) | 1758,127s | 1732,766 | 1783,648 | 2007,1 | 1993,9 | 2033,2 | 1 |
| Track & Field - Marathon (W) | 8010,233s | 7871,285 | 8151,998 | 2045,1 | 2030,5 | 2063,1 | 1 |
| Track & Field - 20km walk (W) | 5101,599s | 5067,330 | 5135,802 | 2027,5 | 2017,8 | 2039,4 | 3 |
| Track & Field - 100m Hurdles (W) | 12,095s | 11,994 | 12,196 | 2022,8 | 2003,0 | 2054,2 | 1 |
| Track & Field - 400m Hurdles (W) | 52,194s | 51,886 | 52,505 | 2018,2 | 1998,1 | 2069,6 | 2 |
| Track & Field - 4*100m relay (W) | 40,983s | 40,088 | 41,893 | 2027,5 | 1984,7 | 2160,8 | 2 |
| Track & Field - 4*400m relay (W) | 193,686s | 190,532 | 196,782 | 2011,0 | 1988,3 | 2060,6 | 1 |
| Track & Field - High jump (W) | 2,099m | 2,063 | 2,135 | 2000,6 | 1982,2 | 2085,2 | 3 |
| Track & Field - Pole vault (W) | 5,146m | 4,878 | 5,422 | 2045,9 | 2013,1 | 2128,0 | 2 |
| Track & Field - Long jump (W) | 7,547m | 7,465 | 7,629 | 1993,0 | 1985,7 | 2013,1 | 3 |
| Track & Field - Triple jump (W) | 15,631m | 15,093 | 16,171 | 2002,9 | 1991,7 | 2094,4 | 1 |
| Track & Field - Discus throw (W) | 78,905m | 76,642 | 81,153 | 2043,4 | 2014,9 | 2089,9 | 3 |
| Track & Field - Hammer throw (W) | 78,49m | 77,266 | 79,700 | 2019,4 | 2008,9 | 2036,9 | 1 |
| Track & Field - Shot put (W) | 22,8m | 22,647 | 22,952 | 2004,8 | 1997,5 | 2013,6 | 3 |
| Track & Field - Heptathlon (W) | 7341,529 | 7122,168 | 7563,400 | 1997,3 | 1983,9 | 2073,4 | 1 |
| Track & Field - 100m (M) | 9,726s | 9,677 | 9,776 | 2019,2 | 1996,8 | 2126,5 | 3 |
| Track & Field - 200m (M) | 19,193s | 18,990 | 19,400 | 2040,4 | 1999,5 | 2121,9 | 1 |
| Track & Field - 400m (M) | 43,009s | 42,617 | 43,401 | 2036,4 | 1991,0 | 2130,5 | 3 |
| Track & Field - 800m (M) | 100,584s | 98,943 | 102,194 | 2052,2 | 1981,3 | 2315,4 | 3 |
| Track & Field - 1500m (M) | 205,178s | 202,899 | 207,432 | 2025,9 | 1991,0 | 2154,9 | 4 |
| Track & Field - 3000m Steeple chase (M) | 468,774s | 462,866 | 474,806 | 2067,5 | 2020,9 | 2158,1 | 3 |
| Track & Field - 5000m (M) | 754,01s | 744,178 | 763,755 | 2033,0 | 1996,4 | 2142,9 | 3 |
| Track & Field - 10000m (M) | 1575,759s | 1572,589 | 1578,987 | 2008,6 | 2002,8 | 2017,2 | 3 |
| Track & Field - Marathon (M) | 7388,47s | 7324,533 | 7452,136 | 2080,3 | 2054,5 | 2112,3 | 2 |
| Track & Field - 20km walk (M) | 4622,507s | 4601,707 | 4643,639 | 2020,9 | 2009,3 | 2036,7 | 3 |
| Track & Field - 50km walk (M) | 12877,216s | 12823,958 | 12930,889 | 2042,4 | 2030,3 | 2056,8 | 2 |
| Track & Field - 110m Hurdles (M) | 12,711s | 12,497 | 12,926 | 2107,6 | 2034,6 | 2235,7 | 2 |
| Track & Field - 400m Hurdles (M) | 46,537s | 46,319 | 46,755 | 2021,6 | 2005,1 | 2044,3 | 2 |
| Track & Field - 4*100m relay (M) | 37,105s | 36,682 | 37,531 | 2054,0 | 2004,7 | 2169,2 | 3 |
| Track & Field - 4*400m relay (M) | 173,383s | 169,462 | 177,285 | 2035,7 | 1972,2 | 2775,3 | 2 |
| Track & Field - High jump (M) | 2,467m | 2,433 | 2,501 | 2027,4 | 1997,8 | 2090,9 | 4 |
| Track & Field - Pole vault (M) | 6,154m | 6,118 | 6,190 | 2001,1 | 1990,8 | 2028,2 | 5 |
| Track & Field - Long jump (M) | 891,454m | 857,991 | 924,691 | 1991,7 | 1965,4 | 2225,7 | 2 |
| Track & Field - Triple jump (M) | 18,495m | 18,163 | 18,826 | 2057,9 | 2007,3 | 2159,1 | 3 |
| Track & Field - Discus throw (M) | 76,022m | 74,030 | 77,975 | 2050,2 | 2016,2 | 2106,5 | 3 |
| Track & Field - Hammer throw (M) | 87,189m | 85,169 | 89,167 | 1992,0 | 1982,2 | 2043,9 | 2 |
| Track & Field - Shot put (M) | 23,833m | 23,178 | 24,477 | 2077,1 | 2036,7 | 2137,6 | 2 |
| Track & Field - Decathlon (M) | 9088,487 | 8930,790 | 9248,429 | 2041,3 | 1994,9 | 2234,2 | 3 |
| WeightLifting - C&J 48kg (W) | 118,77kg | 116,408 | 121,102 | 2017,0 | 2002,2 | 2080,9 | 1 |
| WeightLifting - C&J 53kg (W) | 128,211kg | 125,324 | 131,089 | 2006,6 | 1999,6 | 2042,3 | 1 |
| WeightLifting - C&J 58kg (W) | 140,905kg | 134,718 | 147,042 | 2017,7 | 2001,9 | 2098,7 | 1 |
| WeightLifting - C&J 63kg (W) | 142,836kg | 140,864 | 144,817 | 2021,3 | 2012,0 | 2037,1 | 1 |
| WeightLifting - C&J 69kg (W) | 155,877kg | 149,193 | 162,540 | 2018,7 | 2004,4 | 2063,9 | 1 |
| WeightLifting - C&J 75kg (W) | 161,216kg | 154,475 | 168,068 | 2016,8 | 2003,2 | 2069,6 | 1 |
| WeightLifting - C&J 75pluskg (W) | 185,388kg | 163,540 | 207,413 | 2016,7 | 1998,7 | 2500,6 | 1 |
| WeightLifting - Snatch 48kg (W) | 99,248kg | 93,290 | 105,252 | 2017,9 | 2002,3 | 2090,5 | 1 |
| WeightLifting - Snatch 53kg (W) | 103,364kg | 100,942 | 105,788 | 2007,7 | 2000,7 | 2031,3 | 1 |
| WeightLifting - Snatch 58kg (W) | 111,931kg | 103,754 | 120,154 | 2013,8 | 1999,7 | 2152,2 | 1 |
| WeightLifting - Snatch 63kg (W) | 117,178kg | 114,676 | 119,692 | 2017,4 | 2005,3 | 2049,0 | 1 |
| WeightLifting - Snatch 69kg (W) | 123,685kg | 116,691 | 130,696 | 2014,6 | 1999,4 | 2191,6 | 1 |
| WeightLifting - Snatch 75kg (W) | 127,043kg | 120,637 | 133,453 | 2016,1 | 1999,6 | 2191,6 | 1 |
| WeightLifting - Snatch 75pluskg (W) | 137,713kg | 137,395 | 138,039 | 2004,3 | 2002,6 | 2006,6 | 1 |
| WeightLifting - C&J superheavyweight (M) | 263,793kg | 263,396 | 264,182 | 2006,4 | 2003,2 | 2010,6 | 4 |
| WeightLifting - C&J lightheavyweight (M) | 218,636kg | 212,266 | 224,989 | 2002,4 | 1993,2 | 2490,1 | 5 |
| WeightLifting - C&J lightweight (M) | 198,202kg | 197,042 | 199,386 | 2014,4 | 2002,8 | 2042,4 | 5 |
| WeightLifting - C&J flyweight (M) | 163,053kg | 158,000 | 168,224 | 2024,0 | 1998,2 | 2097,3 | 3 |
| WeightLifting - Snatch superheavyweight (M) | 213,864kg | 208,702 | 218,874 | 2010,3 | 1995,4 | 2119,1 | 5 |
| WeightLifting - Snatch middleheavyweight (M) | 188,093kg | 188,055 | 188,130 | 1999,0 | 1998,4 | 1999,6 | 4 |
| WeightLifting - Snatch lightheavyweight (M) | 187,42kg | 178,280 | 196,455 | 2064,4 | 2007,5 | 2174,6 | 2 |
| WeightLifting - Snatch middleweight (M) | 173,826kg | 173,675 | 173,977 | 2007,0 | 2005,7 | 2008,4 | 4 |
| WeightLifting - Snatch bantamweight (M) | 139,797kg | 137,964 | 141,632 | 2031,0 | 2009,9 | 2064,8 | 5 |
